# Supplementary material for: Memory B Cells Induced by Sputnik V Vaccination Produce SARS-CoV-2 Neutralizing Antibodies Upon Ex Vivo Restimulation
Source: Front Immunol. 2022 Feb 24;13:840707. doi: 10.3389/fimmu.2022.840707 (PMC8907154; doi:10.3389/fimmu.2022.840707)

Supplementary table 1. Participant characteristics

Recipient ID	Sex	Age	COVID-19 symptoms[§]	Vaccination symptoms	
				1st dose	2st dose
1	M	61	-70 to -61[§§]	37.8°C	38.7°C
2	M	69	-53 to -46[§§]	injection site reaction	w/o
3	F	62	w/o	w/o	w/o
4	F	60	w/o	w/o	w/o
5	M	60	w/o	w/o	w/o
6	M	70	w/o	headache	w/o
7	F	66	w/o	w/o	w/o
8	F	54	w/o	w/o	w/o
9	M	66	w/o	w/o	w/o
10	F	43	w/o	w/o	w/o
11	F	61	w/o	w/o	w/o
12	F	52	w/o	w/o	w/o
13	F	25	w/o	w/o	w/o
14	F	46	w/o	w/o	w/o
15	F	43	w/o	w/o	w/o
16	F	51	w/o	w/o	injection site reaction
17	M	42	-86 to -78[§§]	37.8°C	38.7°C
18	M	61	-120 to -110[§§]	37.8°C	w/o
19	F	66	w/o	w/o	w/o
20	F	62	w/o	w/o	w/o
21	F	53	-88 to -71[§§]	w/o	w/o
22	F	53	w/o	37.5°C	37.5°C

[§] mild COVID-19 symptoms (cough, sore throat, headache, runny nose, and muscle ache) were observed on the indicated days.

[§§] PCR-confirmed COVID-19.

Supplementary Figure 1. Study design

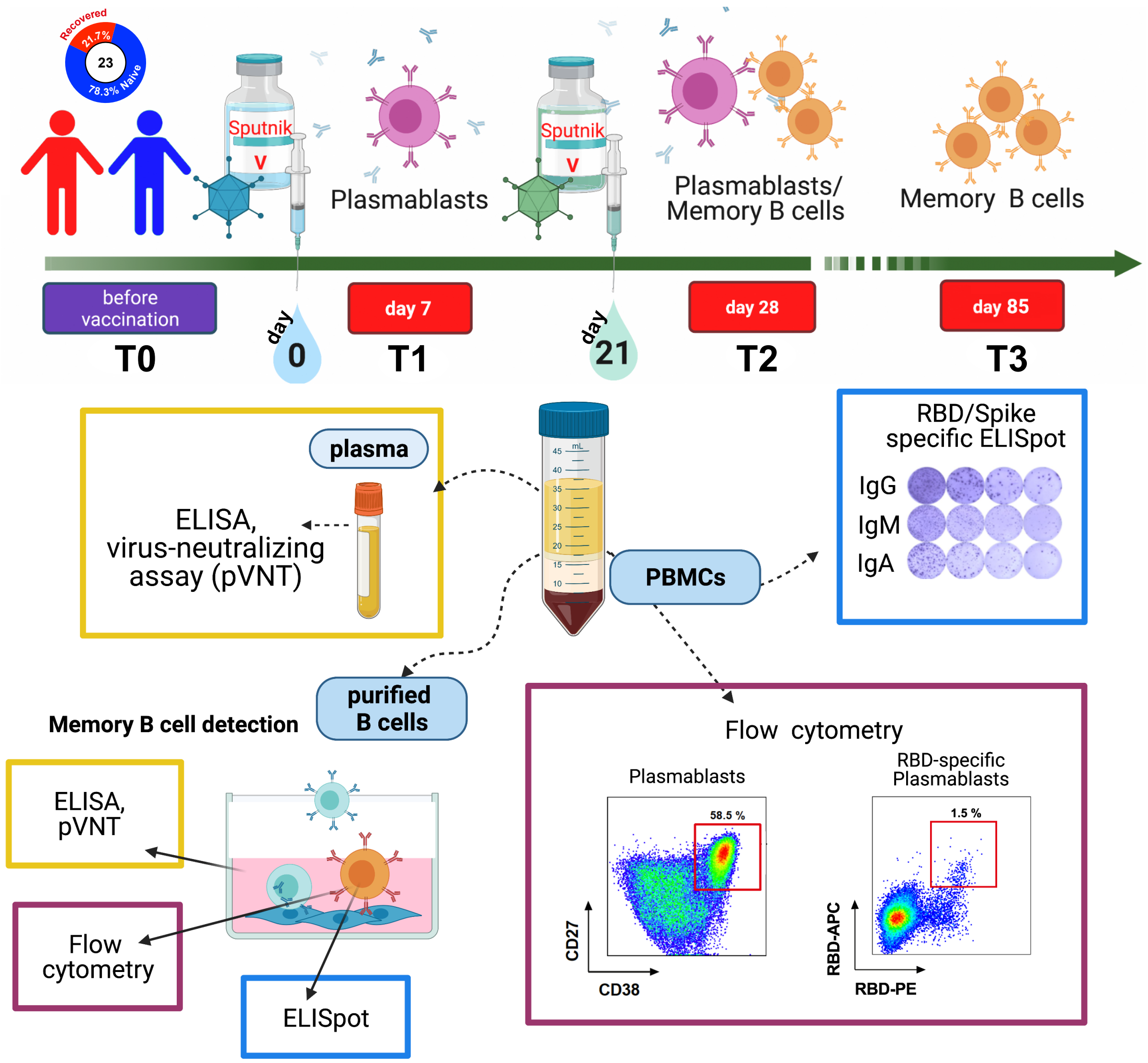

Supplementary Figure 2. IgG antibodies against the receptor-binding domain (RBD) (green bars), and nucleocapsid (N)(black bars) of SARS-CoV-2 at T0 time point just before vaccination. The dotted lines indicate the threshold for anti-RBD (green) and anti-Nucleocapsid IgG (black) positivity.

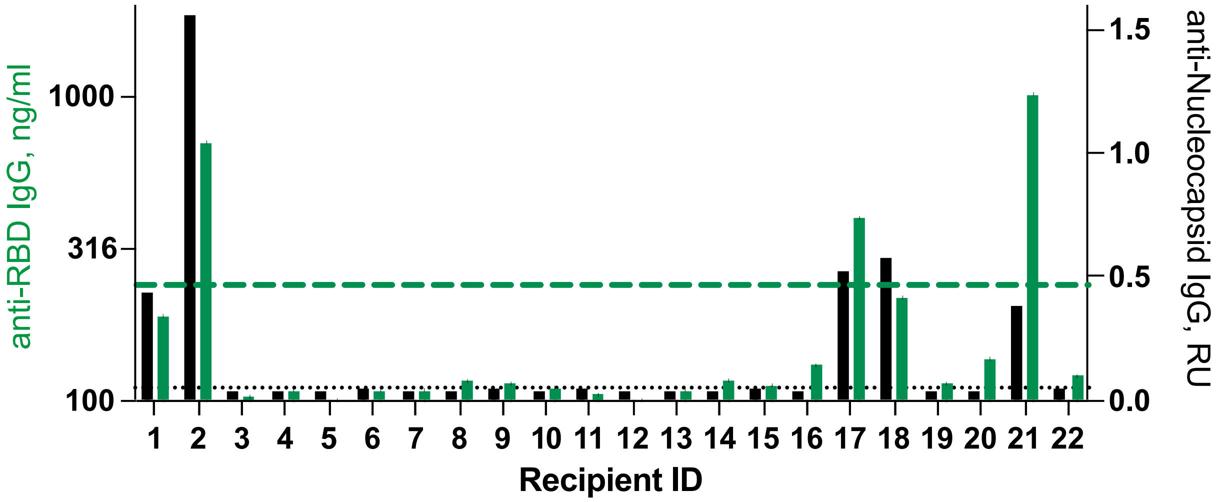

Supplementary Figure 3. Neutralization curves of vaccine-induced sera against pseudotyped virus expressing SARS-CoV-2 WA1 or Beta variant Spike protein. Neutralization activities of sera from naïve (n=17, blue frames) and recovered (n=5, red frames) individuals were measured at T3 time point.

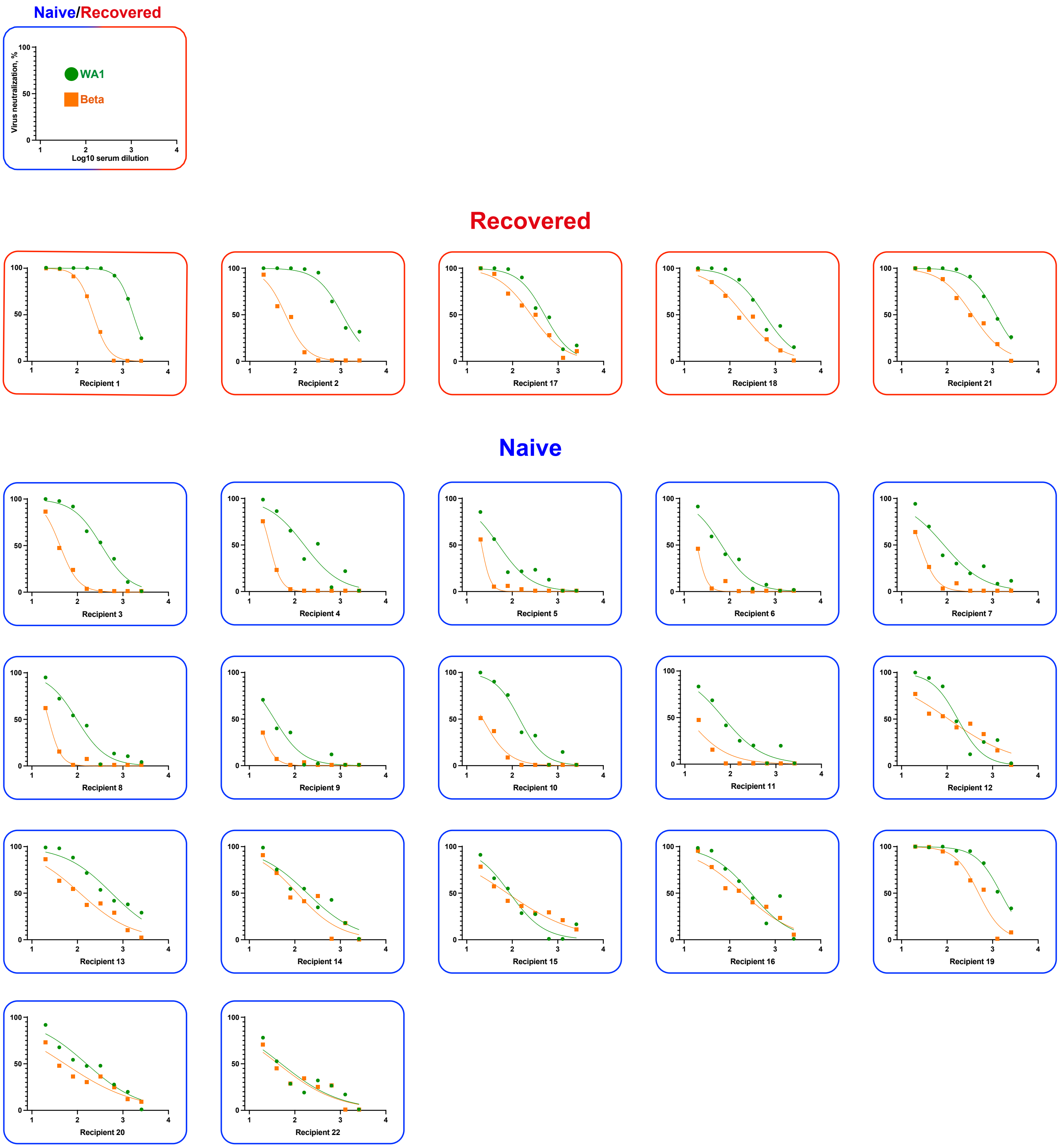

Supplementary Figure 4. Spearman's correlation between virus neutralization (ID_{50}) against WA1 strain and Beta variant at T3 time point for sera from vaccinated individuals

.

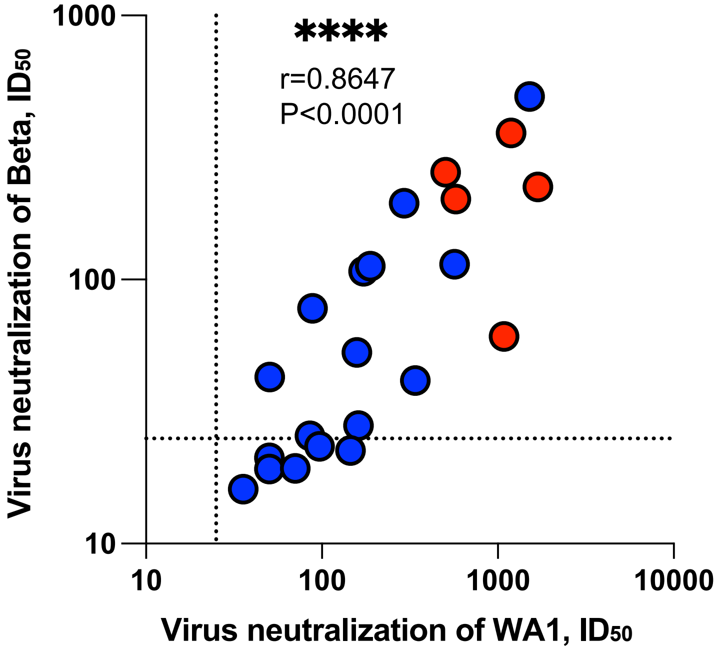

Supplementary Figure 5. Plasmablast dynamics during stimulation of different B lymphocyte subpopulations. The $IgG^{+}CD27^{+}$, $IgM^{+}CD27^{+}$, $IgM^{+}IgD^{+}$ subsets were sorted out from peripheral blood lymphocytes, stimulated *in vitro* with IL-21 and CD40L for indicated days and analyzed using flow cytometry. The results of four separate experiments each with three technical repeats are shown.

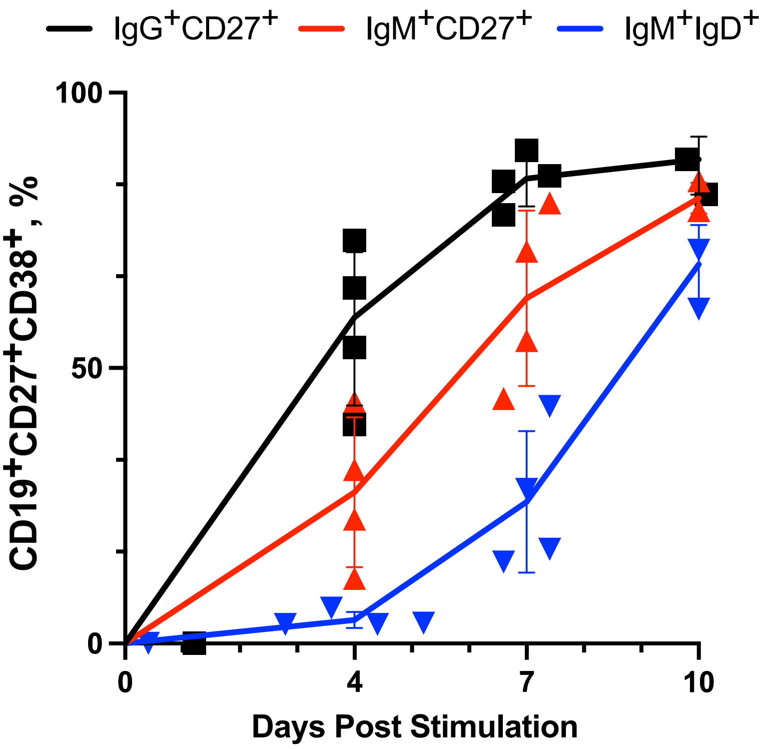

Supplementary Figure 6. Virus-neutralizing activity of antibodies derived from the cultures of CD40L/IL-21-stimulated B cells for individual vaccine recipients against WA1 strain and Beta variant at T2 (upper panel) and T3 (bottom panel) time point. The results of three separateexperiments each with two technical repeats are presented for all vaccine recipients.

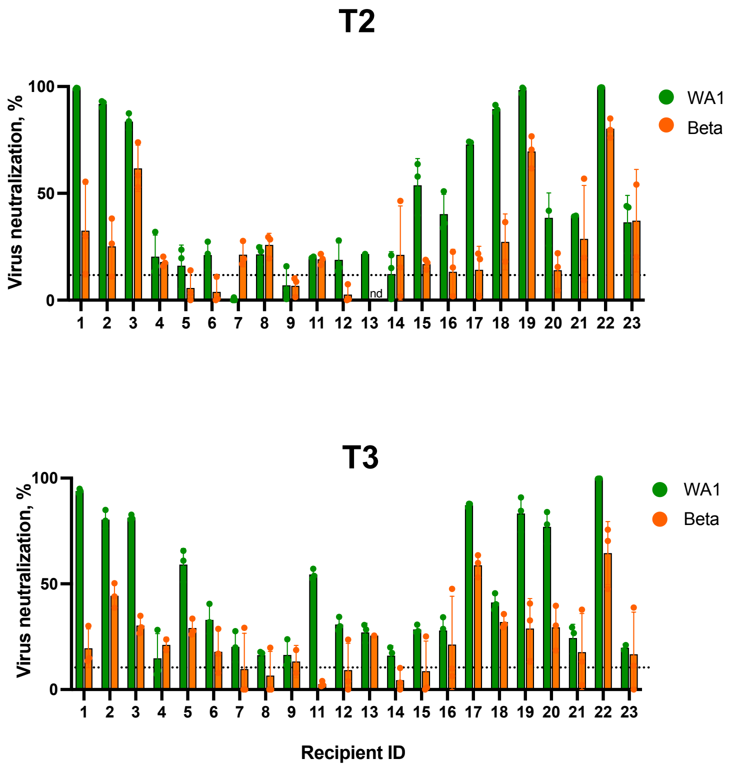

Supplementary Figure 7. Spearman's correlation between virus neutralization of WA1 (%) and the levels of anti-RBD IgG in supernatants of IL-21/CD40L-stimulated B cells obtained from Sputnik V-vaccinated individuals at T2 time point

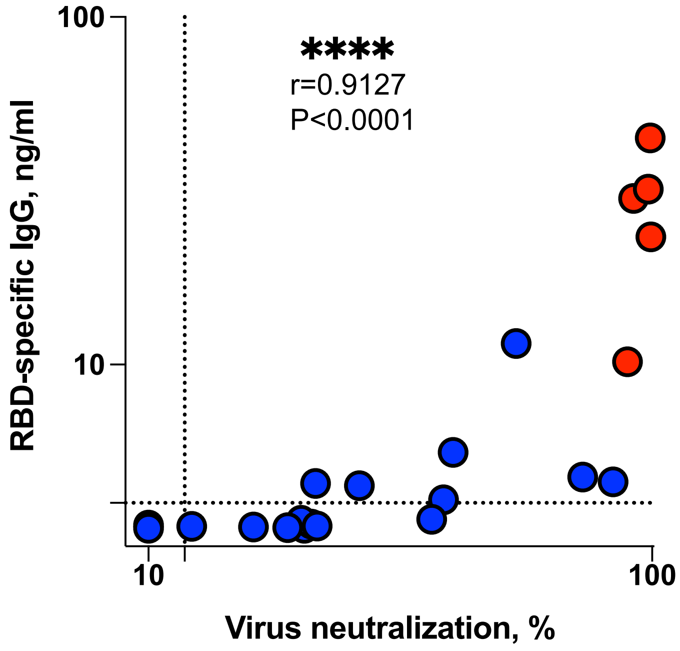

Supplement: Supplementary file 1 [file DataSheet_1.pdf]
